# Editorial: High-Level Adaptation and Aftereffects

**DOI:** 10.3389/fpsyg.2017.00217

**Published:** 2017-02-17

**Authors:** Rocco Palumbo, Stefania D'Ascenzo, Luca Tommasi

**Affiliations:** ^1^Schepens Eye Research Institute, Harvard Medical SchoolBoston, MA, USA; ^2^Department of Psychological Science, Humanities and Territory, “G. d'Annunzio” UniversityChieti, Italy; ^3^Department of Philosophy and Communication, University of BolognaBologna, Italy

**Keywords:** aftereffects, adaptation, high-level adaptation, visual cognition, perception

Adaptation, in sensory and perceptual science, refers to the action of a prolonged exposure of a receiver (at the cellular level a sensory receptor, at the organismic level an animal endowed with sensory organs) to an environmental stimulus. While adaptation of sensory receptors is strictly equivalent to the physiological phenomenon of a reduced electrical response by a receptor following its sustained stimulation, in this Frontiers Research Topic “adaptation” will be conceived at the organismic level, in which a wide spectrum of perceptual effects, mostly known as “aftereffects,” has been observed for more than two thousand years (Aristotle, ca 350 B.C.^1^). Aftereffects refer to those changes in the way a stimulus (the test) is perceived following a prolonged exposure to a previous stimulus (the adaptor). Undoubtly, for a change to be deemed relevant, a comparison must be made between the perception of the test following the presentation of the adaptor vs. the perception of the same test presented in isolation.

Aftereffects following adaptation have been reported for all sensory modalities, and for a growing variety of features in each modality (see Hollins and Favorov, 1994; Schweinberger et al., 2008), although the most investigated domain is certainly vision. Aftereffects have indeed been documented for brightness, color, motion, orientation, size, shape and for various combinations of these visual features (for a review, see Webster, 2015). A general interpretation of aftereffects following adaptation of low-level vision is related to the phenomenon of neural adaptation, which is an extension of sensory receptor adaptation, in that it entails the weakening of electrochemical activity of neural units responsible for processing and communicating the signals corresponding to low-level visual information beyond the level of sensory receptors, in the visual brain (Barlow and Hill, 1963). In other words, the sensory and neural adaptation following sustained stimulation may well provide the basis to explain the change in perceptual appearance that is typical of aftereffects in many low-level visual adaptation phenomena. The fact that there is a strict contingency between sensory and neural change and perceptual outcomes has even referred to perceptual adaptation as the “psychophysicist's microelectrode” (Frisby, 1979), because the behavioral quantification of its nature and dynamics is an important behavioral window on the underlying processes of the visual brain.

Besides the classical aftereffects related to low-level vision, in recent decades perception science has intriguingly escaladed toward high-level adaptation aftereffects, thus paralleling two crucial discoveries in the neurosciences: (i) the perceptual brain is organized hierarchically, with low-level units (and regions) feeding high-level units (and regions) that represent objects (Webster, 2011), and (ii) among units and regions representing objects, some of them are specialized to process objects that have a very important adaptive value for the organism, such as faces (e.g., Webster et al., 2004), bodies (e.g., Winkler and Rhodes, 2005; Palumbo et al., 2013), emotions(e.g., Fox and Barton, 2007), and other complex environmental and social entities (Clifford and Rhodes, 2005; Greene and Oliva, 2010).

Studies focusing on high-level adaptation have involved more and more complex objects of perception, but have also moved in the direction of testing the boundaries of these time-sensitive perceptual (and neural) phenomena on many fronts: (i) does the duration of stimulus exposure influence the ensuing effects in systematic ways? (ii) are the effects contrastive (the test is perceived in the direction opposite to the adaptor) or assimilative (the test is perceived in the same direction as the adaptor)? (iii) are the effects of adaptation confined to a given modality or do they extend crossomodaly? (iv) are the effects merely perceptual, or are they contingent and/or permeable to other processing levels (attention, learning, imagery, language)? (v) does the behavioral evidence fit with data obtained from the neurosciences, and does it provide us with a tenable benchmark to make predictions on and build models of the brain? We believe that replying to all of these questions is both important and far-reaching. It is important because the answers seem to strongly reaffirm the role of perceptual adaptation in the cognitive neurosciences broadly conceived, possibly updating its symbolic status to that of a “psychophysicist's microelectrode and scanner,” to move with the current times (Cziraki et al., 2010). It is also far-reaching because adaptation offers a great window of opportunity to put diverse aspects into contact. Being a time-and-order-sensitive phenomenon, adaptation is akin to many other neural and psychological (even merely methodological) effects that span across multiple research traditions (sensitization, peak shift, priming, serial order effects, anchoring, carry-over effects, etc.), and that still do not have a unifying framework. The usefulness of adaptation to test aftereffects occurring across modalities (e.g., Skuk and Schweinberger, 2013) is an added value to the field of crossmodal perception research. Additionally, even more promising is the understanding of aftereffects occurring across perceptual and conceptual categories and the role of other levels of processing in adaptation (Ghuman et al., 2010; Palumbo et al., 2015), to the point that adaptation stands as a key tool not only for the psychophysicist, but for the cognitive scientist interested in mapping the boundaries between perception and cognition, and in the interplay among representations and processes (Leopold et al., 2001; Storrs, 2015).

We hope that some glimpses of all of these opportunities will be conveyed by this Frontiers Research Topic, which starts with some papers dealing with face aftereffects, a domain of adaptation studies that has grown at a fast pace in the last two decades (see Strobach and Carbon, 2013; Hills, 2013). The first paper, by Zimmer et al. is focused on the effects of variation of the temporal factor (adaptation duration) on the manifestation of facial identity aftereffects and specific time-sensitive components recorded by means of ERPs. The authors highlight the role of exposure duration in evoking the electrophysiological components of face processing, showing that longer adaptation induces enhanced components and a more articulated segregation of the stages of category-, image- and identity-related processing. The paper by Kloth et al. investigates the potential interplay between sex and gaze direction in a gaze adaptation paradigm, concluding in favor of the nature of gaze direction as a natural adaptable category, independent of sex contingencies, and showing how a thorough manipulation of stimulus dimensions in the experimental design of an adaptation paradigm can shed light on the important issue of hierarchy and functional independence in perceptual processing. In the paper by Davidenko et al., the role of attention is carefully examined in an adaptation paradigm that is focused on either the ethnicity or the sex of faces, showing that attention plays no role in the direction of aftereffects, though it has an effect in modulating the speed of response of participants. Finally, the paper by Ross and Palmeri is an overview of the requirements (and the pitfalls) of a biologically- and behaviorally-inspired approach to modeling face processing, that capitalizes on data from face adaptation phenomena, providing valuable directions and theoretical discussion for computational modeling in general.

The following two papers revolve around aftereffects observed for another important high-level entity, the human body. The article by Brooks et al. investigates the differential role of adaptating to one's own or another person's body size (along the dimension of fatness/thinness), on perceived body size aftereffects, concluding (similarly to Kloth et al.'s paper) that body size is a visual feature that can produce adaptation effects *per se*, independently in this case, of any contingency with self vs. other identity of the observed body. In the second paper, Stephen et al., propose a novel methodological paradigm useful to assess body size aftereffects (again, along the dimension of fatness/thinness), factoring in the role of explicit vs. implicit attentional set, and concluding (similarly to Davidenko et al.'s paper) that attention does not play a major role in visual adaptation to body size.

Two further papers delve more into aspects concerning adaptation to emotional features. In the paper by Wincenciak et al., which could also be listed alongside the two previous papers, the authors make use of adaptation to human bodies, although they do so to assess whether the emotional information conveyed by whole-body actions is contingent upon the identity of the actors performing those actions. They demonstrate that identity appears to act as a determinant in the modulation of emotional action aftereffects, albeit in a graceful fashion, with stronger and longer-lasting aftereffects in the case of same identities, as compared to the case of different identities, which evoke aftereffects in a weaker and short-lived fashion. The paper by Palumbo et al. moves far from the domain of strictly biological entities, in the attempt to evaluate the effect of adaptation to the overall emotional valence of complex images. Carefully excluding faces from the set of images employed, the authors show that aftereffects can be evoked following adaptation to complex scenes, but have an assimilative rather than a contrastive nature, suggesting that visually perceived emotional valence is grounded on brain mechanisms eluding the phenomenon of neural adaptation.

The final two papers in the Research Topic are also based on less common adaptation paradigms, and demonstrate both the extent of the field and the many potential bridges to other fields. Miyoshi et al., provide evidence that a long adaptation to pictures reduces the strength of priming across disparate domains (images and words), suggesting the action of conceptual factors in mediating the effects, and the major role of exposure duration in explaining experience-driven phenomena such as priming and adaptation. Finally, the paper by Michel offers a brief review of the literature on prism adaptation, a century-old method consisting in visually shifting the entire visual field by wearing prismatic lenses. Although the effects of prism adaptation are primarily sensorimotor (thus constituting a valuable tool for rehabilitation of neglect patients) a growing body of research has investigated cognitive aftereffects following this treatment, and the paper offers important insights on the continuity (or better the discontinuity) between perception, action and higher cognitive processes, and on the ties between adaptation and plasticity.

To conclude, the papers in this Research Topic offer a wide cross-section of the research being carried out in the field of high-level adaptation, a complex territory that has now a well-established body, but also branches to other territories, demonstrating a great potential for further developments. Aftereffects, once a phenomenon confined to perception science, have indeed stood the test of time as a tool for studying perception, but have also assumed a wider relevance, as they now allow us to better understand the relationships between perception and other levels of representation (action, imagery, cognition, emotion, language), to behaviorally gauge the workings of the brain, and to build more robust computational models.

## Author contributions

All authors listed, have made substantial, direct and intellectual contribution to the work, and approved it for publication.

### Conflict of interest statement

The authors declare that the research was conducted in the absence of any commercial or financial relationships that could be construed as a potential conflict of interest.

## Footnotes

^1^Aristotle (ca 350 B.C.). *Parva Naturalia*.
